# Using simulation to aid trial design: Ring-vaccination trials

**DOI:** 10.1371/journal.pntd.0005470

**Published:** 2017-03-22

**Authors:** Matt David Thomas Hitchings, Rebecca Freeman Grais, Marc Lipsitch

**Affiliations:** 1 Center for Communicable Disease Dynamics, Harvard T. H. Chan School of Public Health, Boston, Massachusetts, United States of America; 2 Epicentre, Paris, France; Imperial College London, UNITED KINGDOM

## Abstract

**Background:**

The 2014–6 West African Ebola epidemic highlights the need for rigorous, rapid clinical trial methods for vaccines. A challenge for trial design is making sample size calculations based on incidence within the trial, total vaccine effect, and intracluster correlation, when these parameters are uncertain in the presence of indirect effects of vaccination.

**Methods and findings:**

We present a stochastic, compartmental model for a ring vaccination trial. After identification of an index case, a ring of contacts is recruited and either vaccinated immediately or after 21 days. The primary outcome of the trial is total vaccine effect, counting cases only from a pre-specified window in which the immediate arm is assumed to be fully protected and the delayed arm is not protected. Simulation results are used to calculate necessary sample size and estimated vaccine effect. Under baseline assumptions about vaccine properties, monthly incidence in unvaccinated rings and trial design, a standard sample-size calculation neglecting dynamic effects estimated that 7,100 participants would be needed to achieve 80% power to detect a difference in attack rate between arms, while incorporating dynamic considerations in the model increased the estimate to 8,900. This approach replaces assumptions about parameters at the ring level with assumptions about disease dynamics and vaccine characteristics at the individual level, so within this framework we were able to describe the sensitivity of the trial power and estimated effect to various parameters. We found that both of these quantities are sensitive to properties of the vaccine, to setting-specific parameters over which investigators have little control, and to parameters that are determined by the study design.

**Conclusions:**

Incorporating simulation into the trial design process can improve robustness of sample size calculations. For this specific trial design, vaccine effectiveness depends on properties of the ring vaccination design and on the measurement window, as well as the epidemiologic setting.

## Author summary

The urgency, as well as the logistical and sometimes ethical challenges of clinical trials for interventions during epidemics of emerging diseases prompts the need for novel designs and analytic strategies. The successful use of a novel cluster-randomized ring-vaccination trial to test an Ebola vaccine in Guinea raises the general question of what circumstances would favour the use of trials of similar design and how the properties of the population, the vaccine and the trial would influence the necessary sample size and the expected results. We present a generalized transmission dynamic model for a ring vaccination trial to address these questions. This work is an example of the general phenomenon that mechanistic, transmission-dynamic simulations can aid in the design and interpretation of intervention trials for infectious diseases, when the trial itself can have non-obvious effects on transmission dynamics that may not be fully captured by effect- and sample-size calculations for noncommunicable diseases.

## Introduction

The West African Ebola epidemic highlighted the need to identify a range of trial designs to evaluate vaccine effects rapidly, efficiently and rigorously during emerging disease outbreaks. The ring-vaccination trial approach employed in the *Ebola ça suffit* trial in Guinea is one innovative approach [1], which produced valuable evidence that the vaccine could prevent Ebola infection [2]. Other approaches considered include individual randomization and a stepped-wedge design [3, 4]. In such trials it is difficult to estimate the likely effect of an infectious disease intervention because of indirect effects, and this issue is compounded by complex trial design. Sample size calculations are based on group-level quantities such as intervention effect and are therefore potentially inaccurate. By creating a transmission dynamic model for a ring vaccination trial, we show that we can make sample size calculations based on disease characteristics and individual intervention efficacy. With this framework in place we are then able to examine the estimated vaccine effect and sample size under a range of assumptions about the properties of the vaccine, the trial, and the study population.

Although the only implementation of the ring trial design has been in Guinea during the Ebola epidemic, lessons can be learned and extended to other diseases and contexts. Here, we examine the tail end of an epidemic of a disease with a latent and asymptomatic phase with effective contact tracing to illustrate a more widely-applicable set of findings. In particular, we use baseline parameters values consistent with Ebola in West Africa in 2014–6, but we vary several assumptions over broader ranges than those occurring in the *Ebola ça suffit* trial, with the aim of being relevant to a range of potential future situations.

## Methods

### Ring vaccination trial

The simulation is based on a stochastic, susceptible-exposed-infectious-detected-removed-vaccinated (SEIDRV) model for individual disease events, and it represents progression of the disease in a small cluster (henceforth ‘ring’) with homogeneous mixing. The ring represents both contacts and contacts of contacts so the assumption of homogeneous mixing is a simplifying assumption, which we can relax by modelling ‘contacts’ and ‘contacts of contacts’ as separate compartments with the highest transmission among the contacts. New cases arise through direct contact between an infectious individual and a susceptible individual within the ring, and through external infectious pressure, denoted by F, which is constant and fixed for all members of the ring. Members of the ring undergo surveillance by the study team, meaning that infectious individuals are detected and isolated with a daily probability p_H_, ending their infectious period. We assume in the baseline scenario that detection rate in the trial is equivalent to routine surveillance, reflecting the fact that the trial doesn’t interrupt or enhance disease control efforts. If infectiousness ends naturally, individuals can no longer be detected.

A ring is enrolled into the trial when a case is detected through routine surveillance. This first detected case is defined as the index case for the purposes of the trial, but may or may not be the true index case of the outbreak in the ring. Once a ring enters the trial all its members are randomly assigned to immediate vaccination (on day 1) or delayed vaccination (on day 22). In the baseline scenario we assume no ineligibility or non-consent, so that all susceptible and exposed individuals in the ring are vaccinated, and that there is no heterogeneity or administrative delay affecting the day of vaccination.

The mechanism of the vaccine in an individual is as follows: multiplicative leaky efficacy [5] increases linearly from 0 to VE (set at baseline to be 0.7) over a period of D_ramp_ days following vaccination, after which there is no change in efficacy over the study period [6].

### Statistical analysis

Statistical analysis of the trial is based on cumulative incidence in the rings by end of follow-up and a 95% confidence interval is calculated and reported [7]. The required sample size to test a vaccine effect with 80% power is based on a difference in cumulative incidence [8], using parameters output by a simulated trial with 15,000 rings. We chose this analysis method because of the existence of simple closed-form sample size and vaccine efficacy formulae. Because both arms receive the vaccine, cases that contribute towards the cumulative incidence in each arm are only counted during a window in which the immediate arm is presumed to be protected by the vaccine, and the delayed arm is not protected. The window length is set to 21 days, equal to the vaccination delay between the arms. Because the disease has an asymptomatic phase and the vaccine has a ramp-up period during which it is not fully efficacious, the window starts at 16 days, the sum of the average asymptomatic period length and D_ramp_, in an attempt to exclude cases in the immediate arm who were infected before they were fully protected by the vaccine. We did not explicitly implement clustering in the simulation, instead assuming that transmission dynamics in all rings are independent. However, clustering of cases within rings arises naturally due to dependent happenings. We measure this clustering using the intracluster correlation coefficient (ICC), calculated as per Shoukri et al [9], adjusting for the covariate of trial arm and accounting for variable ring size where appropriate.

In conducting the statistical analysis we assume full knowledge of the vaccine mechanism, and that cases are only included if they are detected before their infectious period ends, and their symptoms appeared during the window.

For additional details on the disease transmission model, ring initiation, and analysis of the trial see the supplementary appendix.

### Choice of parameters

Table 1 shows the parameters used in the model, their meanings, values under baseline assumptions, and references or justifications.

**Table 1:** Meaning and choice of parameters.

Parameter	Meaning	Default value	Reference
R_eff_	Average detected secondary infections from each infected individual in a susceptible population, in the presence of background case detection	0.61	Calibration to 2% detected monthly attack rate with background case detection, from a single index case
Mean (latent)	Mean latent period length (days)	9.31	[10]
SD (latent)	Standard deviation of latent period length (days)	5.28	[10]
Mean (infectious)	Mean infectious period length (days)	7.41	[10]
SD (infectious)	Standard deviation of infectious period length (days)	3.24	[10]
P_BH_	Daily probability of detection before start of trial	0.2	Mean of 5 days to hospitalization [11]
P_H_	Daily probability of detection after start of trial	0.2	Baseline assumption, corresponding to no change in detection from background rate during the trial
VE	Individual vaccine efficacy	0.7	Baseline assumption [6]
D_ramp_	Days after vaccination until vaccine efficacy reaches VE	6	Baseline assumption [2]
D_start_	First day of counting cases	16	Assumption (based on sum of vaccine ramp-up period and mean incubation period)
F	External force of infection	0	Assumption (following rationale of a ring vaccination trial designed to place vaccine in areas of high local transmission)
m	Size of a ring	50	Baseline assumption of *Ebola ça suffit* trial [1]

Table of parameter values and meanings, and references for those parameters which were chosen using the literature

In order to align this model with the presumed context of the *Ebola ça suffit* trial, we modelled an entirely susceptible study population at the end of an epidemic, so that R_eff_ has fallen to below one due to behaviour change. To calibrate the model, we set R_eff_ to reproduce a monthly detected attack rate of 2% when starting from one infected individual in a ring of 50 unvaccinated susceptible individuals, in the presence of case detection at a rate p_BH_.

## Results

Under the baseline parameter assumptions listed above, the sample size necessary in each arm to achieve 80% power to detect a difference in cumulative incidence between the two arms is 89 rings, each containing 50 individuals, making a total of 8,900 study participants. This trial would on average return a total vaccine effect estimate of 69.81%, with average 95% CI (28.5, 87.2).

### Determinants of vaccine effectiveness estimate

Under baseline parameters in this model, the median total vaccine effect calculated from performing 100 trials with 89 rings in each arm was 70%. This value should include direct and indirect effects, so we would expect it to exceed the direct effect of 70%. However, while direct effects begin immediately, indirect effects are only important in the second generation of preventable cases onwards. There are cases in this generation that occur in the case-counting window because R_eff_ is small and the window duration is not much longer than a typical disease generation (17 days), so the indirect effects are small.

**Fig 1:**
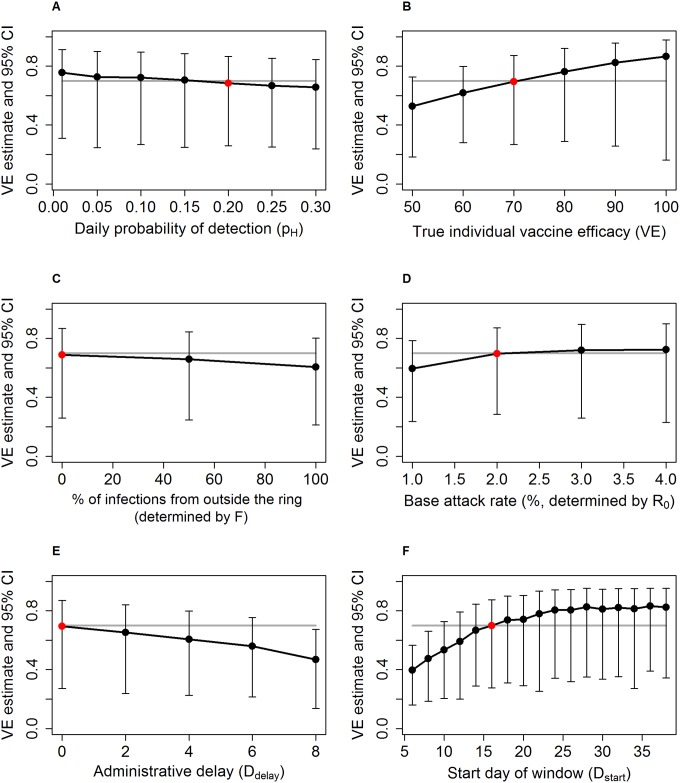
Estimate of vaccine effect by trial design, disease, vaccine and population characteristics. Median point estimate of vaccine effect and 95% confidence interval derived from 100 trials with 80% power to detect vaccine effect shown against: (left to right, top to bottom) A: daily probability of detection, B: true individual vaccine efficacy, C: proportion of infections from outside the ring, D: baseline attack rate in the unvaccinated population, E: administrative delay in vaccination, and F: start day of case-counting window. In each panel, the VE estimate corresponding to the baseline parameter set is highlighted in red, and the grey line represents the individual vaccine efficacy of 70%. All other parameters are set at the baseline values.

Fig 1 shows the effect of six variables on the point estimate of vaccine effect: daily probability of detection, true individual vaccine efficacy, proportion of infections from outside the ring, baseline attack rate in the unvaccinated population, administrative delay in vaccination, and start day of case-counting window.

Firstly, if there is enhanced surveillance in both arms of the trial leading to more rapid isolation of infectious cases (p_H_>p_BH_), this will modestly reduce effectiveness estimates (Fig 1A). Secondly, as individual vaccine efficacy properties increase the estimated vaccine effect increases (Figs 1B and S1). Thirdly, the percentage of infections from within the ring shows a weak negative association with the estimate of vaccine effect (Fig 1C). While the magnitude of indirect effects is modest as discussed above, they are almost negligible when most infections are from outside the ring, because preventing infections within the ring does not confer as much protection to susceptible individuals. The increase in vaccine effect with higher attack rate seen in Fig 1D is driven by the increase in indirect vaccine effects in the immediate arm. Finally, delay between ring formation and vaccination means that by the beginning of the time window the vaccine has had less time to prevent cases in the immediate arm. Thus the reduction in incidence in the immediate arm does not reflect the true effect of the vaccine and the vaccine effect estimate is reduced (Fig 1E).

A major determinant of the effect estimate is the choice of time window in which to count cases, as seen in Fig 1F. Not surprisingly, starting the window too early reduces the estimated effects because it includes a period of time during which the vaccine cannot affect the incidence of cases becoming symptomatic–many cases becoming symptomatic on day 8, for example, will have been infected by the index case prior to isolation, or will have been infected by a contact on (say) day 3, before the vaccine had time to induce protection.

Starting the window later than the baseline of 16 days allows the trial to capture later generations in the chain of transmission, from a vaccinated person to another vaccinated person. This increases the vaccine effect estimate as it includes indirect effects. One might expect to see that starting the window too late would reduce effect estimates because it would include a period when the delayed group was also protected by the vaccine. This does not appear to be the case, at least up to a start time of 35 days (Fig 1F)–see the supplementary material for an explanation of this phenomenon.

### Determinants of sample size

**Fig 2:**
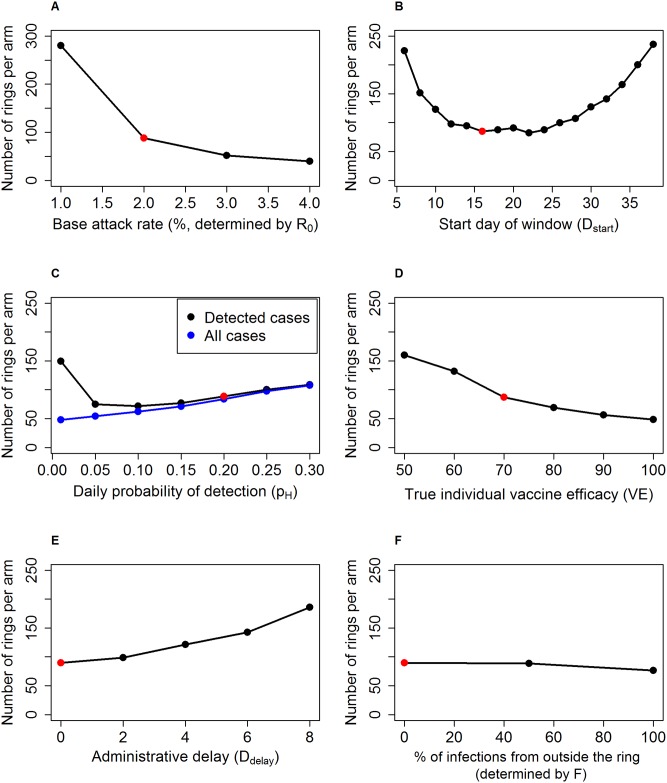
Required sample size by trial design, disease, vaccine and population characteristics. Number of rings per arm required to achieve 80% power to detect a difference in cumulative incidence between the two arms against: (left to right, top to bottom) A: baseline attack rate in unvaccinated population, B: start day of case-counting window, C: daily probability of detection, D: true individual vaccine efficacy, E: administrative delay in vaccination, and F:proportion of infections from outside the ring. In Fig 2C, sample sizes are shown for VE estimates based on only detected cases (black) and on all cases (blue). In each panel, the sample size estimate corresponding to the baseline parameter set is highlighted in red. All other parameters are set at the default values.

Fig 2 shows the effect of the same six variables on the required sample size: baseline attack rate in unvaccinated population, start day of case-counting window, daily probability of detection, true individual vaccine efficacy, administrative delay in vaccination, and force of external infection.

The effect of each parameter on the sample size can be understood through its effect on one or more of the three factors that determine the power of this trial: the number of events, how they are distributed between the two arms, and the level of clustering of cases within rings. Respectively these factors are represented by the attack rate in the controls, the cumulative incidence difference between the arms, and the intracluster correlation coefficient (ICC) [8].

Variables that decrease the incidence rate in the controls and cases will decrease the power because for the same sample size the trial will observe fewer events. The baseline detected attack rate among unvaccinated individuals is a simple example of such a parameter (Fig 2A). Two other parameters act on the overall incidence in the trial. Firstly, making the start of the case-counting window later decreases incidence in both arms because with R_eff_<1 the incidence is on average declining, so across all rings in the trial the number of cases decreases over the follow-up period (Fig 2B). Secondly, the case detection decreases detected incidence rate at both extremes (Fig 2C). When case detection is high, transmission chains are interrupted by case isolation and the true incidence decreases. When case detection is low, many cases die or recover before they can be detected and consequently the detected incidence decreases.

Variables that make the two arms of the trial appear more different will increase the power of the trial as the ability to differentiate between them is increased, and Fig 1 identifies such variables. Vaccine characteristics, in particular vaccine efficacy (Fig 2D), are simple examples of such a parameter, since the immediate arm receives greater protection against disease compared to the delayed arm. Changes to two other parameters increase the incidence difference in this way, as explained above: reducing the delay between ring formation and vaccination (Fig 2E) and starting the case-counting window earlier (Fig 2B).

The effect of the timing of starting to count cases thus reflects two opposing forces on the sample size: it decreases sample size by increasing the incidence difference, and it increases sample size by decreasing the overall incidence. When the window is early, the former of these effects dominates as seen by the increase in sample size for early time windows in Fig 2B. When the window is late, the latter effect dominates, as seen by the increase in sample size for late time windows in the same figure.

Finally, the level of clustering within rings inflates the sample size, because more clustering means that each individual case provides less information. It is often not intuitive to predict the direction in which a parameter will cause the ICC to change, and in many cases the ICC is not sensitive to the parameter. One exception is the infection from outside the ring (Fig 2F). The most significant effect of introducing external infection and reducing within-ring transmission is to make infection probability for one individual within a ring independent from the infection prevalence within the same ring. This reduces clustering in incidence (making it more Poisson-like), thus reducing the ICC and the necessary sample size.

The width of the confidence intervals is affected in the same way by the three variables described above. In particular, low incidence in either arm, high ICC and a small incidence difference between the arms all lead to a wider confidence interval. The formula for the confidence interval is different from the formula used to make the power calculation, so the trends do not completely align because the size of the effect of each of the three factors is different for the confidence interval and the sample size.

For an investigation of the sensitivity of the total vaccine effect estimate and sample size to other parameters in the model, see the supplementary material. For an interactive tool to explore the sensitivity of the trial parameters, see https://matthitchings.shinyapps.io/ShinyApps/.

## Discussion

The ring-vaccination, cluster-randomized design has two key strengths that make it a good candidate when disease transmission exhibits spatiotemporal variation. Firstly, by including members of the study population who are contacts of cases, the trial preferentially selects those at higher risk of disease acquisition, leading to an increase in efficiency while preserving false-positive rate through randomization. Indeed, when a vaccine with 0% efficacy was tested in our simulations the false positive rate was maintained at 5%. Secondly, even those study subjects who are randomized to delayed vaccination are theoretically in close contact with the study team meaning that individuals from the source population who are at the highest risk are followed closely and benefit from the trial even in the absence of vaccination [12].

In addition, vaccination of clusters when they arise allows for gradual inclusion, meaning that this design is appropriate when logistical constraints make immediate vaccination of all participants impossible or inappropriate. In this respect it is similar to a stepped-wedge cluster trial, in which prespecified clusters within the study population are vaccinated in a random order. Although we have not made a direct comparison in this study, Bellan et al [13] showed that the stepped-wedge design is underpowered when the incidence is declining because it cannot prioritize the vaccine for those at highest risk. The ring vaccination design, on the other hand, is inherently risk-prioritized because all study participants should be at higher risk than the general population.

All trials should be correctly powered in order to avoid erroneous rejection of an efficacious vaccine. For a trial design with several complexities such as the one presented here, a sophisticated approach to sample size calculation is merited. A standard approach to sample size calculation for this trial would involve specifying the attack rate among the controls, the desired effect of the vaccine on the population level, and the ICC. In the context of a serious epidemic, these parameters are unlikely to be estimated with certainty; for example, the ICC requires cluster-level data to be estimated accurately. The ICC is an important parameter in designing cluster-randomized trials, yet in the absence of data it is often assumed to be 0.05. In our simulations the range of ICCs observed was 0.01–0.04, suggesting that the value of this uncertain parameter should not always be assumed to be fixed at 0.05. Therefore, the modelling approach replaces assumptions about these cluster-level quantities with assumptions about population-level parameters and disease characteristics, which are more likely to be available through analysis of data from the outbreak.

A second advantage of the modelling approach is that, based as it is on a simulating the transmission of disease within a trial, it is possible to explore the impact of parameters describing the design of the trial and the properties of the disease. The added detail gained from specifying the disease model allowed us in this study to identify some key issues with the design that are worth considering.

Firstly, as seen in Fig 2C, increasing case-finding efficiency above background rate has a negative impact on power, as fast isolation of cases in both arms leads to an overall decrease in cases observed by the trial. In future trials it is worth considering if there are alternative or composite endpoints, if the disease in question permits, that can be used to allow for efficacy estimates while maintaining close follow-up.

Secondly, a key design consideration in the delayed-arm ring-vaccination trial is when to count cases. An intuitively appealing approach is to place the window so that the immediate arm is receiving full protection and the delayed arm none. This should in theory minimize bias caused by misclassification of unvaccinated individuals as vaccinated and vice-versa. While this placement achieves nearly maximal power, it does not maximize the VE estimate. Indirect effects that are important later in time increase the VE estimate for later time windows, while at the same time declining incidence within each ring decreases power for later time windows.

Finally, the above point draws attention to the fact that caution is required when interpreting the VE estimate produced by the trial. As seen in Fig 1, many parameters that are not characteristics of the vaccine can influence the estimated effect. Whether this is due to misclassification (for example, when the time window is too early) or due to indirect effects (for example, when the attack rate is high enough to cause long transmission chains), the context of the trial should be taken into account when interpreting the VE estimate. While in the baseline scenario the trial appears to correctly estimate the individual efficacy, this is the result of misclassification and indirect effects cancelling each other out. This claim is supported by the fact that the median VE estimate falls below the individual-level vaccine efficacy when most or all infections are from outside the ring (Fig 1C) and indirect effects are negligible.

The focus of this model was to explore parameters within each ring and understand how they affect the quality of data coming from the trial. As a result, we did not consider the wider context of the population disease dynamics, and in particular how and when the rings arise. For example, we calibrated R_eff_ to a secondary attack rate in a cluster was 2%, which is not necessarily comparable to the monthly cumulative incidence in the population. If transmission takes place mainly in clusters then population cumulative incidence could be somewhat lower than cluster secondary attack rate, increasing the efficiency of a ring-vaccination trial relative to a stepped-wedge cluster trial or individual RCT. Linking this model to a model of disease within the general population would allow us to make direct comparisons to other trial designs such as the stepped-wedge cluster trial and the individually-randomized trial investigated elsewhere [13, 14], but it would require detailed information about the nature of clustering of the disease in this context, and for simplicity we focused on the within-ring dynamics only.

As with every model, there are limitations to these simulation results. The strength of the modelling approach compared with a standard approach is that it better estimates the parameters on which the sample size depends. However, some of the model parameters might still be uncertain in a situation in which such a model might be useful. For example, we may have limited information about the characteristics of a disease, in particular its latent and incubation period, and its R_eff_. The simulation results are dependent on these assumptions, and so they cannot be used at the very outset of epidemic, or else they risk being highly inaccurate. Even at the end of the West African Ebola epidemic, there were no more than four or five reliable estimates of the latent and infectious periods of EVD, and indeed there is perhaps evidence that our understanding of the natural history of the disease remains limited [15]. In addition, we have considered only the simplest method of analysis for the trial–a comparison of attack rates between the two arms after correction for clustering of cases within rings. More sophisticated methods, including time-to-event analyses incorporating ring-level random effects, as performed in the *Ebola ça suffit* trial, would have somewhat different sample size requirements. However, we believe that the trends seen here would be similar for other methods, because the VE estimates returned by various methods will be similar for a rare outcome [5]. In building the model we made some simplifying assumptions, and although we tested the robustness of the results to these assumptions (see supplementary material) it is possible that a more sophisticated model would provide more accurate results, particularly if superspreading events are not rare in this study population.

For a vaccine trial in an epidemic, when the level of indirect effects is hard to predict, power calculations can be sensitive to parameters about which very little is known. Simulations such as these can be important aids in understanding a range of values for these parameters before a trial is carried out, and thus ensuring that the trial has sufficient power to detect an efficacious vaccine. In this trial, a finding significantly different from the null likely indicates one or more types of vaccine efficacy at the individual level, but the magnitude of the effect and the power to detect the effect will vary across settings.

## Supporting information

S1 File Appendix. Additional detail on methods, including disease transmission model and simulation and analysis of trial, and results including an explanation of why estimated vaccine effect doesn’t decrease with later time windows, and sensitivity of vaccine effect estimate and sample size to other parameters in the model.
(DOCX)

S1 Code R code. Code to estimate required sample size and vaccine effect from a ring vaccination trial for a chosen set of parameters. The computations in this paper were run on the Odyssey cluster supported by the FAS Division of Science, Research Computing Group at Harvard University.
(DOCX)

S1 Fig Log incidence rate in simulated trial populations. Simulated log incidence rate of detected disease in the trial, in the immediate arm (black circles) and delayed arm (blue circles), with linear fit in the immediate arm (black line) and piecewise linear fit in the delayed arm (blue line). The change in rate in the delayed arm corresponds to the direct effect of the vaccine. Circles represent means over 15,000 simulations.
(TIFF)

S2 Fig Estimate of vaccine effect by trial design, vaccine and population characteristics. Median point estimate of vaccine effect and 95% confidence interval derived from 100 trials with 80% power to detect vaccine effect shown against: (left to right, top to bottom) A: post-exposure vaccine efficacy, B: days to maximum individual vaccine efficacy, C: average vaccine coverage in a ring, D: range in ring size, and E: ring size. In each panel, the VE estimate corresponding to the baseline parameter set is highlighted in red, and the grey line represents the individual vaccine efficacy of 70%. All other parameters are set at the baseline values.
(TIFF)

S3 Fig Required sample size by trial design, vaccine and population characteristics. Number of rings per arm required to achieve 80% power to detect a difference in cumulative incidence between the two arms against: (left to right, top to bottom) A: post-exposure vaccine efficacy, B: days to maximum individual vaccine efficacy, C: average vaccine coverage in a ring, D: range in ring size, and E: ring size. In each panel, the sample size estimate corresponding to the baseline parameter set is highlighted in red. All other parameters are set at the default values.
(TIFF)

S4 Fig Estimate of vaccine effect and required sample size by disease latent period. Relationship between the start day of case-counting window and A: the median point estimate of vaccine effect derived from 100 trials with 80% power to detect vaccine effect, and B: required sample size for 80% power to detect vaccine effect, for a disease with a short, baseline and long latent period. In Fig S4A, the grey line represents the individual vaccine efficacy of 70%. All other parameters are set at the baseline values.
(TIFF)

S5 Fig Estimate of vaccine effect and required sample size by disease infectious period. Relationship between the start day of case-counting window and A: the median point estimate of vaccine effect derived from 100 trials with 80% power to detect vaccine effect, and B: required sample size for 80% power to detect vaccine effect, for a disease with a short, baseline and long infectious period. In Fig S5A, the grey line represents the individual vaccine efficacy of 70%. All other parameters are set at the baseline values.
(TIFF)
