# Supplementary material for: Using simulation to aid trial design: Ring-vaccination trials
Source: PLoS Negl Trop Dis. 2017 Mar 22;11(3):e0005470. doi: 10.1371/journal.pntd.0005470 (PMC5378415; doi:10.1371/journal.pntd.0005470)
Supplement: S1 File — Additional detail on methods, including disease transmission model and simulation and analysis of trial, and results including an explanation of why estimated vaccine effect doesn’t decrease with later time windows, and sensitivity of vaccine effect estimate and sample size to other parameters in the model. (DOCX) [file pntd.0005470.s001.docx]

**S1. Appendix**

S1.1. Disease transmission model

To simulate the spread of a disease within a small (m=50) community of individuals who have close contact with each other (henceforth a “ring”), we used a stochastic, compartmental model with six compartments: susceptible, susceptible vaccinated, exposed, infectious, isolated, and removed (either recovered or dead). The time step was one day, and all processes such as infection, disease progression, etc. are discretized to occur at the end of a particular day. We assumed that individuals become infectious when symptoms appear, meaning that the latent period and incubation period are concurrent (WHO. http://www.who.int/mediacentre/factsheets/fs103/en/). Each individual in the susceptible compartment has a daily force of infection from two sources: externally from individuals not contained within the ring, and internally from individuals within the ring. The former is denoted by a fixed, constant hazard F/day, and the latter has hazard equal to βI/day, where β is the transmission rate constant and I is the number of infectious individuals in the ring. An individual who becomes infected is placed in the exposed compartment, where they spend a number of days determined by a gamma distribution, with mean 9.31 days and variance 27.92 (days^2^)[1]. At the end of the latent period, the individual is moved into the infectious compartment, where they spend a number of days determined by an independent gamma distribution with mean 7.41 days and variance 10.49 (days^2^)[1]. While an individual is in the infectious compartment, they have a per-day probability of being detected and isolated, p_H_. The act of isolation immediately ends their infectiousness, meaning that case detection stops transmission. If they reach the end of the infectious period without being detected, they are placed into the removed category, at which point they are no longer infectious. In this model we have not allowed for a *post mortem* period of infectiousness, nor the possibility of sexual transmission among those recovered, nor of asymptomatic infections.

In the baseline scenario we assume a simple ring structure: specifically, that all individuals in the ring mix homogeneously with all other individuals and with the index case, and that all rings are of the same size. To relax the first assumption we assume that the ring is made up of an index case, ‘contacts’, and ‘contacts of contacts’, represented by separate compartments with 1, 7 and 43 individuals respectively. Within each compartment individuals mix homogeneously, and the index case exerts infectious pressure on contacts only, while contacts exert infectious pressure on other contacts, and contacts-of-contacts. Contacts-of-contacts are not assumed to cause further infection, translating to an assumption that transmission chains of longer than three are neglibile. With R_0_ significantly less than one and the time window of 21 days, we believe this is a good assumption. To relax the second assumption we assume that ring size is uniformly distributed on a given range.

S1.2. Ring vaccination trial details

Initially, we considered a vaccine whose only effect was pre-exposure prophylaxis; in initial runs we assumed the vaccine had no effect if given to a person who was exposed but not yet infectious, an assumption we later relaxed. When included, post-exposure vaccine effects were modelled as follows: when a vaccinated, latently infected subject leaves the exposed class, he will move straight to the removed class with probability p_PEP_. In the baseline scenario all members of the ring are eligible and consenting, an assumption that we can relax by vaccinating only a proportion of the individuals in the ring. In this case, only those who are vaccinated are included in the analysis.

To initiate rings, we simulated the following steps: One infected individual (not counted in the m=50) is infected, and the length of his infectious period is drawn from the gamma distribution. As he progresses through his infectious period, susceptible members of the ring can be infected with daily probability 1 – e^-βI + F^. In addition, the index case can be detected and isolated with daily probability p_BH_. For baseline simulations we set p_BH_=0.2, meaning that it takes on average 5 days to detect and isolate an infected individual[2]. If he is detected before his infectious period ends, he is rendered non-infectious by isolation, and he becomes the index case for a ring, which proceeds as described in the Methods section of the main text. If he is not detected before recovering or dying, he is effectively invisible to trial investigators, so he is not counted as an index case, and the simulation is terminated and repeated again, without counting the “invisible” case as part of the study sample.

S1.3. Trial simulation and analysis

The sample size calculation includes an inflation factor (1 + ρ*(m-1)), where ρ is the intracluster correlation coefficient (ICC). The cumulative incidence of detected EVD cases is recorded in each arm of the trial, and the vaccine effectiveness is estimated as VE_est_ = (1 – CI_imm_/CI_del_)*100, where CI_imm_ is the cumulative incidence in the immediate arm and CI_del_ is the cumulative incidence in the delayed arm. Since we expect the event to be rare, the calculation of vaccine effect will be approximately equal to the measure using the hazard ratio[3]. As we are assuming no vaccine ineligibility or refusal, this quantity estimates the combination of the direct and indirect effect of the vaccine[4]. In order to output the likely estimate of vaccine effect derived from this trial, we perform the trial 100 times at the required sample size calculated above, and we report the median vaccine effectiveness estimate from these 100 trials, and 95% confidence intervals calculated using the average standard deviation observed in the 100 trials[5]. All simulation was performed using R[6].

**Results**

Understanding why vaccine effect doesn’t decrease with later time windows

Before vaccination, incidence in both arms is decreasing at the same exponential rate, and thus in proportion to each other. The effect of vaccination is to increase the rate of decline in the immediate arm by interrupting potential transmission chains. The difference between the two arms increases as indirect effects come into play, until the delayed arm receives vaccination. The effect of vaccination in the delayed arm is to increase the rate of decline so that it is equal to the rate in the immediate arm. This explains why the VE estimate doesn’t decrease for later time windows; the incidence in the delayed arm doesn’t ‘catch up’ with that in the immediate arm, it merely ‘keeps pace’ when the vaccine begins to have an effect. Figure S1 shows, on the log scale, the change in incidence rate decline in the delayed arm that happens around day 30-35, or 9-14 days after vaccination. After that, the two lines are parallel on the log scale, meaning that they are declining in proportion and so the VE estimate, which is based on the cumulative incidence ratio, doesn’t change appreciably (cumulative incidence is almost proportional to incidence because cumulative incidence is low and thus not saturating).

Figure S1: Simulated log incidence rate of detected disease in the trial, in the immediate arm (black circles) and delayed arm (blue circles), with linear fit in the immediate arm (black line) and piecewise linear fit in the delayed arm (blue line). The change in rate in the delayed arm corresponds to the direct effect of the vaccine. Circles represent means over 15,000 simulations.

Effect of other parameters on vaccine effect estimate and sample size

Figure S2 shows the effect of five variables on the point estimate of vaccine effect: post-exposure vaccine efficacy, days to maximum individual vaccine efficacy, vaccine coverage, range in ring size, and ring size.

Figure S2: Median point estimate of vaccine effect and 95% confidence interval derived from 100 trials with 80% power to detect vaccine effect shown against: (left to right, top to bottom) A: post-exposure vaccine efficacy, B: days to maximum individual vaccine efficacy, C: average vaccine coverage in a ring, D: range in ring size, and E: ring size. In each panel, the VE estimate corresponding to the baseline parameter set is highlighted in red, and the grey line represents the individual vaccine efficacy of 70%. All other parameters are set at the baseline values.

Increasing post-exposure efficacy increases the estimated total vaccine effect (Figure S2A), because vaccination of exposed individuals prevents them becoming infectious, thus reducing incidence in the immediate arm. If the case-counting window is set to start at 16 days, increasing the time to maximum vaccine efficacy decreases the estimated effect (Figure S2B) because individuals in the immediate arm are not fully protected for longer. The other three variables appear to have little effect on the total vaccine effect.

Figure S3 shows the effect of the same five variables on the required sample size.

Figure S3: Number of rings per arm required to achieve 80% power to detect a difference in cumulative incidence between the two arms against: (left to right, top to bottom) A: post-exposure vaccine efficacy, B: days to maximum individual vaccine efficacy, C: average vaccine coverage in a ring, D: range in ring size, and E: ring size. In each panel, the sample size estimate corresponding to the baseline parameter set is highlighted in red. All other parameters are set at the default values.

As in the main text, the effect of each variable on sample size can be understood through its effect on overall incidence, incidence difference and ICC. As seen in Figure S2, increasing post-exposure efficacy and decreasing days to maximum efficacy both increase the estimated vaccine effect, resulting in a corresponding decrease in the required sample size (Figure S3A and S3B).

The next two variables act primarily through the ICC. When vaccine coverage is not perfect a random number of individuals in each ring is vaccinated. Analysis is restricted to those who are vaccinated, meaning that ring size is variable, leading to an increase in ICC[7]. This corresponds to an increase in the ICC and an increase in the sample size (Figure S3C). Introducing ring size variability with a uniform distribution also increases the ICC but to a lesser degree, leading to a very moderate increase in sample size with increasing ring size variation (Figure S3D). Finally, increasing ring size has no effect on the sample size, because it doesn’t change the vaccine effect estimate or the dynamics of the disease within the ring (Figure S3E).

Changing the ring structure to model contacts and contacts of contacts separately so that transmission occurs separately from the index case to contacts, and from contacts to contacts-of-contacts induces a small downward bias in the vaccine effect estimate (from 70% to 66%), due to fewer observed tertiary cases in both arms and smaller indirect effects. However, it has no material effect on the required sample size (sample size 86 rings per arm for baseline assumptions). Since there is very little ongoing transmission in the study population, whether the secondary case occurs among the contacts or contacts of contacts has little effect on subsequent spread of the disease. We acknowledge that this model doesn’t allow for the presence of highly connected individuals in a community, but in this setting when R_eff_<1 due to behaviour change, we believe that infectivity of individuals is likely to be limited and that superspreading events are rare. In this instance, within-cluster structure has little effect on the power of a trial[8].

Finally, we varied characteristics of the disease natural history to understand how the optimal counting window changes with the latent and infectious period length. We chose a ‘short’ and ‘long’ latent period (average length 4.5 and 18 days respectively, with a corresponding change in variance), and a ‘short’ and ‘long’ infectious period (average length 3.7 and 15 days respectively, with a corresponding change in variance), and varied the start day of the case-counting window for each of these cases. The results are plotted in Figures S4 and S5, alongside the baseline scenario.

Figure S4: Relationship between the start day of case-counting window and A: the median point estimate of vaccine effect derived from 100 trials with 80% power to detect vaccine effect, and B: required sample size for 80% power to detect vaccine effect, for a disease with a short, baseline and long latent period. In Figure S4A, the grey line represents the individual vaccine efficacy of 70%. All other parameters are set at the baseline values.

As the latent period increases, the case-counting window that minimises power becomes later to cover the period of time in which susceptible individuals in the immediate arm are protected by the vaccine and those in the delayed arm are not (Figure S4B). Similarly, for a fixed window the vaccine effect estimate is larger when the latent period is shorter because the indirect effect is larger (Figure S4A). When the latent period is longer and more variable, the vaccine effect estimate is biased downwards because there are more cases counted in the immediate arm that were infected before vaccination. This corresponds to an increase in the required sample size (Figure S4B).

Figure S5: Relationship between the start day of case-counting window and A: the median point estimate of vaccine effect derived from 100 trials with 80% power to detect vaccine effect, and B: required sample size for 80% power to detect vaccine effect, for a disease with a short, baseline and long infectious period. In Figure S5A, the grey line represents the individual vaccine efficacy of 70%. All other parameters are set at the baseline values.

In contrast, changing the length of the infectious period doesn’t change the optimal window (Figure S5B). There are two primary reasons for this: firstly, because of active detection of cases the length of the infectious period is not as different between the three diseases as it would be in the absence of detection; secondly, the window starting around 16-20 days still captures the period of time in which susceptible individuals in the immediate arm are protected by the vaccine and those in the delayed arm are not, regardless of the infectious period length. There is a small increase in tertiary cases counted in the trial when the infectious period is shorter, leading to a slight increase in the estimated vaccine effect (Figure S5A) and a slight decrease in the required sample size (Figure S5B). These results suggest that the *a priori* choice of case-counting window of the sum of vaccine ramp-up and average latent period is a good one, so long as these two parameters are known with some certainty.

**Bibliography**

1. Althaus CL, Low N, Musa EO, Shuaib F, Gsteiger S. Ebola virus disease outbreak in Nigeria: Transmission dynamics and rapid control. Epidemics. 2015;11:80-4. doi: 10.1016/j.epidem.2015.03.001. PubMed PMID: 25979285.

2. Team WHOER. Ebola Virus Disease among Male and Female Persons in West Africa. N Engl J Med. 2016;374(1):96-8. doi: 10.1056/NEJMc1511045. PubMed PMID: 26736010.

3. Smith PG, Rodrigues LC, Fine PEM. Assessment of the protective efficacy of vaccines against common disease using case-control and cohort studies. Int J Epidemiol. 1984;13(1):87-93.

4. Halloran ME, Longini IM, Struchiner CJ. Design and analysis of vaccine studies. New York: Springer; 2010. 387 p.

5. O'Neill RT. On sample sizes to estimate the protective efficacy of a vaccine. Stat Med. 1988;7:1279-88.

6. Team RDC. R: A language and environment for statistical computing. Vienna, Austria: R Foundation for Statistical Computing; 2008.

7. Kerry SM, Bland JM. Unequal cluster size for trials in English and Welsh general practice: implications for sample size calculations. Stat Med. 2001;20:377-90.

8. Staples PC, Ogburn EL, Onnela JP. Incorporating Contact Network Structure in Cluster Randomized Trials. Sci Rep. 2015;5:17581. doi: 10.1038/srep17581. PubMed PMID: 26631604; PubMed Central PMCID: PMCPMC4668393.
