# Supplementary material for: Using simulation to aid trial design: Ring-vaccination trials
Source: PLoS Negl Trop Dis. 2017 Mar 22;11(3):e0005470. doi: 10.1371/journal.pntd.0005470 (PMC5378415; doi:10.1371/journal.pntd.0005470)
Supplement: S1 Code — Code to estimate required sample size and vaccine effect from a ring vaccination trial for a chosen set of parameters. The computations in this paper were run on the Odyssey cluster supported by the FAS Division of Science, Research Computing Group at Harvard University. (DOCX) [file pntd.0005470.s002.docx]

**S1 Code.** R code to estimate required sample size and vaccine effect from a ring vaccination trial for a chosen set of parameters. The computations in this paper were run on the Odyssey cluster supported by the FAS Division of Science, Research Computing Group at Harvard University.

# Updated Mar 7th 2017 by Matt Hitchings

# By Marc Lipsitch, Harvard T.H. Chan School of Public Health

# Ring vaccine simulation comparing two ring delays. Running this script will, for a given set

# of parameters, calculate required sample size for 80% power to detect difference in cumulative incidence

# between the two arms. Based on a trial of this size, it will then calculate estimated vaccine effect and

# 95% confidence interval.

simulatetrial<-function(pdhosp, VEmax, startcount,nsim,Reff,extfoi_numerator, admin_delay=0, VEtake=6, PEPVE=0, Vuptake=1, window_length=21) {

# Input parameters

# pdhosp: Per-day probability of detecting and isolating an infectious individual

# VEmax: After vaccination and a ramp-up period, individual leaky efficacy reaches VEmax, reducing hazard of infection by a factor (1-VEmax)

# startcount: Counting from isolation of index case, the first day on which cases are counted towards vaccine effect estimate

# nsim: Number of rings simulated in each arm

# Reff: Reff, used to set the per-day hazard of infection for an individual, beta (see below)

# extfoi_numerator: Numerator of the external force of infection, to determine the per-day hazard of infection from outside the ring

# To calibrate Reff and extfoi_numerator, we set them to reproduce a given secondary attack rate starting from a single infectious individual

# To fix the proportion of cases from outside the ring, we set Reff to produce x% SAR in the absence of external infection, then set extfoi_numerator

# to produce 2x% SAR, e.g. to fix 50% of infections from outside the ring.

# admin_delay: Time between identification of an index case and vaccination of the immediate ring. The same delay is applied to the delayed ring

# VEtake: Number of days for vaccine efficacy to reach VEmax, during which it increases linearly

# PEPVE: Post-exposure efficacy, for a vaccinated, exposed individual, the probability that it moves from the exposed compartment directly to the

# removed compartment

# Vuptake: The probability that an individual who is offered a vaccine receives the vaccine

# window_length: Delay between vaccination of the immediate and delayed rings

library(Hmisc)

# Daily probability of detection and isolation before the trial start - i.e. detection of index case and its secondary cases

pdhosppre<-0.2

# Adjust startcount and endcount to account foro administrative delay

startcount<-startcount+admin_delay

endcount<-startcount+window_length

# Number of individuals in a cluster (not including the index case)

m<-50

# Total length of follow-up

endfu<-84

# Times of vaccination

tvacs =admin_delay+c(1,1+window_length)

# Disease natural history

maxnE<-21 #maximum days incubation/latent

maxnI<-14 #maximum days infectious

meannI<-7.41 #mean days infectious (in absence of detection and isolation)

meannI_detected<-2.94 # Mean infectious period in presence of daily probability detection 0.2

nSV<-endfu # days post vaccine to track people's susceptibility

extfoi<-extfoi_numerator/100/endfu #foi per capita from outside the ring - default to 1% over the full course of observation (so divide by endfu)

beta<-Reff/m/meannI_detected # daily hazard of infection of an I

nSV<-endfu # days post vaccine to track people's susceptibility

# Parameters for gamma distribution of incubation and infectious periods

inc_shape<-3.04

inc_rate<-0.33

inf_shape<-5.29

inf_rate<-0.71

# Make vectors of conditional probability for whether individuals go to

# next stage of infection

# I've specified the maximum length of each period, so the last entry of the

# vector is 1

inc_probs<-c(rep(0,maxnE-1),1)

inf_probs<-c(rep(0,maxnI-1),1)

for (index in 1:(maxnE-1)) {

inc_probs[index]<-(pgamma(index,inc_shape,inc_rate) - pgamma(index-1,inc_shape,inc_rate))/(1-pgamma(index-1,inc_shape,inc_rate))

}

for (index in 1:(maxnI-1)) {

inf_probs[index]<-(pgamma(index,inf_shape,inf_rate) - pgamma(index-1,inf_shape,inf_rate))/(1-pgamma(index-1,inf_shape,inf_rate))

}

# Vaccine efficacy

# Starts protecting on day VEstart, ramps up linearly over VEtake days to VEmax

VEstart<-1

VES<-c(rep(0,VEstart),(VEmax/VEtake)*(1:VEtake), rep(VEmax,endfu-VEstart-VEtake)) #Vaccine efficacy vs infection each day after vaccination

VEI<-0 #reduced infectiousness if any of vaccinated person who becomes infected (before or after vaccination)

betav<-beta*(1-VEI)

# A function that takes a vector and outputs the indices of the non-zero

# elements, with duplicates for elements that are greater than 1

vectortoindices<-function(vec) {

indices<-which(vec>0)

output<-vector()

for (index in indices) {

for (count in 1:vec[index]) {

output<-c(output,index)

}

}

output

}

# CORE FUNCTIONS

#A time-advancing function to slide people along the conveyor,

# possibly switching from end of state OLD to beg of state NEW

tplus<-function(old,new){

c(tail(old,1),head(new,-1))

}

# A function to randomly pick, for each exposed and infected individual,

# whether they remain in that class or move to the next class.

random_periods<-function(old,probabilities){

ones_switching<-rbinom(length(old),old,probabilities)

ones_staying<-old-ones_switching

num_switching<-sum(ones_switching)

period_lengths<-vector()

if (num_switching>0){

period_lengths<-vectortoindices(ones_switching)

}

list(out1=num_switching,out2=ones_staying,out3=period_lengths)

}

# A function to take the vector of hospitalized individuals and update a data

# frame with info on when they were hospitalized and when they became infectious

# This will only work if subjects can only be hospitalized during their

# infectious period, which is a reasonable assumption

hosptodayinf<-function(daysinfhosp,tohosp,t){

# Each entry of tohosp represents the number of individuals hospitalized

# on that day, which is the day of their infectious period. For each entry

# make a new vector that contains the day of hospitalization (measured in

# time since trial start) and the day of symptom onset

for (index in 1:length(tohosp)) {

if (tohosp[index]>0) {

for (subject in 1:tohosp[index]){

# Day of hospitalization is t, day of symptom onset is t-index+1

temp<-data.frame(dayinf=t-index+1,dayhosp=t)

daysinfhosp<-rbind(daysinfhosp,temp)

}

} else {}

}

daysinfhosp}

# # advance time and progress state, possibly incorporating PEPVE

update<-function(state,pdhosp,time)

{

x<-state

# Advance unvaccinated exposed and infected, taking into account random incubation/

# infectious periods

list[incu_numswitch,incu_stay,inculengths]<-random_periods(x$EU,inc_probs)

newEU<-tplus(0,incu_stay)

list[infu_numswitch,infu_stay,infulengths]<-random_periods(x$IU,inf_probs)

newIU<-tplus(incu_numswitch,infu_stay)

newRU<-x$RU+infu_numswitch

# Advance vaccinated exposed and infected, taking into account random incubation/

# infectious periods

list[incv_numswitch,incv_stay,incvlengths]<-random_periods(x$EV,inc_probs)

newEV<-tplus(0,incv_stay)

newSV<-tplus(0,x$SV) # vaccinated susceptibles move en masse to being vaccinated for one more day

list[infv_numswitch,infv_stay,infvlengths]<-random_periods(x$IV,inf_probs)

IVPrev<-rbinom(1,incv_numswitch,PEPVE) #prevention by PEP of progression

# from last day of EV

newIV<-c(incv_numswitch-IVPrev,head(infv_stay,-1)) #else progress to IV[1]

newRV<-x$RV+infv_numswitch+IVPrev

x$EU<-newEU

x$IU<-newIU

x$RU<-newRU

x$SV<-newSV

x$EV<-newEV

x$IV<-newIV

x$RV<-newRV

# This is to record the lengths of incubation and infectious periods, both

# as a check and as a potentially interesting outcome

inc_lengths<-c(inculengths,incvlengths)

inf_lengths<-c(infulengths,infvlengths)

# Vaccinate - this section will work only if vaccination is on a fixed day tvac for everyone, and happens only once.

if (time==tvac){

# Include vaccine refusal/ineligibility

vacS<-rbinom(1,x$SU,Vuptake)

x$SV<-c(vacS,rep(0,endfu-1))

x$SU<-x$SU-vacS

vacE<-rbinom(maxnE,x$EU,Vuptake)

x$EV<-vacE

x$EU<-x$EU-vacE

vacI<-rbinom(maxnI,x$IU,Vuptake)

x$IV<-vacI

x$IU<-x$IU-vacI

x$RV<-rbinom(1,x$RU,Vuptake)

x$RU<-x$RU-x$RV

} else{}

# Infect

foi<-extfoi+beta*sum(x$IU)+betav*sum(x$IV)

incU<-rbinom(1,x$SU,1-exp(-foi))

incV<-rbinom(nSV,x$SV,(1-exp(-foi))*(1-VES))

x$EU[1]<-incU

x$EV[1]<-sum(incV)

x$SU<-x$SU-incU

x$SV<-x$SV-incV

#hospitalize - assume this ends their infectivity

tohosp<-rbinom(maxnI,x$IU,pdhosp)

x$IU<-x$IU-tohosp

tohospv<-rbinom(maxnI,x$IV,pdhosp)

x$IV<-x$IV-tohospv

x$H<-x$H+sum(tohosp)+sum(tohospv)

# Get info about day infected and hospitalized for those hospitalized

tohosptot<-tohosp+tohospv

daysinfhosp<-data.frame(dayinf=vector(),dayhosp=vector())

if (sum(tohosptot)>0) {

daysinfhosp<-hosptodayinf(daysinfhosp,tohosptot,time)

# Also want to record the length of the time they spent being infectious, to see what the effect of

# hospitalization is on infectious duration

# If they were hospitalized on the same day as they became infectious, that means that their infectious

# period was 1 day, so add 1 to the difference between dayhosp and dayinf

inf_lengths<-c(inf_lengths,daysinfhosp$dayhosp-daysinfhosp$dayinf+1)

}

list(out1=x,out2=daysinfhosp,out3=inc_lengths,out4=inf_lengths)}

#Data extraction functions

#summary stats

detcases<-function(firstday,lastday)

#this counts only detected cases whose symptoms appear in the time window

{sum((Zdays$dayinf>=firstday) & (Zdays$dayinf<=lastday))}

detplusprevcases<-function (firstday,lastday) #detected cases -- those that are hospitalized (assume if you know about them you hospitalize them)

#plus those left over in the I group on the last day of monitoring

{Z$ht[min(84,lastday)]-Z$ht[firstday]+Z$it[min(84,lastday)]-Z$it[firstday]}

detplusrecplusprevcases<-function (firstday,lastday) #the larger group those that were hospitalized or recovered or prevalent(counted on the day of recovery)

{detplusprevcases(firstday,min(84,lastday))+Z$rt[min(84,lastday)]-Z$rt[firstday]}

checksum <-function(Z){(max(Z$nt)==min(Z$nt)) & min(Z$nt)==m+1}

# END CORE FUNCTIONS

# Initialize vectors to store results of each ring

SAR<-vector()

trueSAR<-vector()

botharm_totals<-vector()

botharm_allcases<-vector()

cat("Daily probability of case detection: ", pdhosp, "\n")

cat("Maximum VES: ",VEmax, "\n")

cat("Counting cases from day",startcount,"to day",endcount,"\n\n")

for(ivac in 1:2){

cat("\n")

tvac<-tvacs[ivac]#set day of ring vaccination for this condition

cat("tvac=",tvac,"\n")

totals<-vector()

firstmonth<-vector()

allcases<-vector()

incubation_periods<-vector()

infectious_periods<-vector()

numtrials<-0

numsims<-0

# Not every simulation will lead to a simulated trial because the index case might not be detected before its

# infectious period ends, so only record those that do, and go until we've done nsim simulated trials

while (numtrials<nsim) {

numsims<-numsims+1

#initialize one ring simulation

stateinit<-list(SU=m,SV=rep(0,nSV),EU=c(rep(0,maxnE-1),1),EV=rep(0,maxnE),IU=rep(0,maxnI),IV=rep(0,maxnI),RU=0,RV=0,H=0) #start with a single E

time=-1

dotrial<-0

#set it going until a case is detected or until the epidemic runs out

while (sum(stateinit$EU)+sum(stateinit$IU)>0) {

list[stateinit]<-update(stateinit,pdhosppre,time)

if (stateinit$H>0) {

# if a case is detected, go ahead with the trial

dotrial<-1

numtrials<-numtrials+1

break

}

}

if (dotrial==1) {

# If there are no exposed or infectious individuals when the case is detected, and no external infection,

# no need to run a simulation because there will be no cases.

if ((sum(stateinit$EU)+sum(stateinit$IU)==0) && (extfoi_numerator==0)) {

totals<-c(totals,0)

firstmonth<-c(firstmonth,0)

allcases<-c(allcases,0)

} else {

# Run the simulation, keeping any exposed and infectious individuals from the pre-trial simulation,

# and replacing all detected/recovered individuals with new susceptibles

newsusceptibles=m+1-sum(stateinit$EU)-sum(stateinit$IU)

state<-list(SU=newsusceptibles,SV=rep(0,nSV),EU=stateinit$EU,EV=rep(0,maxnE),IU=stateinit$IU,IV=rep(0,maxnI),RU=0,RV=0,H=0)

time=1

st<-rep(0,endfu)

et<-rep(0,endfu)

it<-rep(0,endfu)

rt<-rep(0,endfu)

ht<-rep(0,endfu)

nt<-rep(0,endfu)

Z<-data.frame(st,et,it,rt,ht,nt)

Zdays<-data.frame(dayinf=vector(),dayhosp=vector()) #records day of start of infectious period for all cases

# Record incubation and infectious periods, and days on which cases arose

onesiminflength<-vector()

onesiminclength<-vector()

inclengths<-vector()

inflengths<-vector()

daysinfhosp<-vector()

for (time in 1:endfu){

Z$st[time]<-sum(state$SU)+sum(state$SV)

Z$et[time]<-sum(state$EU)+sum(state$EV)

Z$it[time]<-sum(state$IU)+sum(state$IV)

Z$ht[time]<-state$H

Z$rt[time]<-state$RU+state$RV

Z$nt[time]<-sum(state$SU)+sum(state$SV)+sum(state$EU)+sum(state$EV)+sum(state$IU)+sum(state$IV)+state$RU+state$RV+state$H

onesiminclength<-c(onesiminclength,inclengths)

onesiminflength<-c(onesiminflength,inflengths)

Zdays<-rbind(Zdays,daysinfhosp)

list[state,daysinfhosp,inclengths,inflengths]<-update(state,pdhosp,time)

}

# Record incubation and infectious periods

incubation_periods<-c(incubation_periods,onesiminclength)

infectious_periods<-c(infectious_periods,onesiminflength)

# Record number of detected cases in the time window

totals<-c(totals,detcases(startcount,endcount))

# Record number of detected cases in the first month after index cases isolation

firstmonth<-c(firstmonth,detcases(1,31))

# Record all cases, whether detected or not, in the time window

allcases<-c(allcases,detplusrecplusprevcases(startcount,endcount))

}

} else {}

}

# Number of cases in both arms, used to estimate ICC

botharm_totals<-c(botharm_totals,totals)

botharm_allcases<-c(botharm_allcases,allcases)

# Overall attack rate in each arm

SAR[ivac]<-mean(totals)/m

trueSAR[ivac]<-mean(allcases)/m

cat("detected cases first month",mean(firstmonth)*100/m,"%\n") #detected SAR in first month

cat("detected cases during followup",SAR[ivac]*100,"%\n") #detected SAR in followup

cat("all cases during followup",trueSAR[ivac]*100,"%\n") #actual SAR

}

# Sample size using a binomial test for proportions

ssu<-(1.96+0.84)^2 * (SAR[1]*(1-SAR[1]) + SAR[2]*(1-SAR[2]))/(m*(SAR[1]-SAR[2])^2) # Outputs per-arm sample size in rings, based on detected cases

truessu<-(1.96+0.84)^2 * (trueSAR[1]*(1-trueSAR[1]) + trueSAR[2]*(1-trueSAR[2]))/(m*(trueSAR[1]-trueSAR[2])^2) # Outputs per-arm sample size in rings, based on true cases

# ICC calculation with covariance adjustment is from Shoukri et al

botharm_totals_sq<-botharm_totals^2

K<-2*nsim

# Covariance-adjusted ANOVA estimator of ICC

MSB<-1/(K-1) * (sum(botharm_totals_sq)/m - sum(botharm_totals)^2/(m*K))

MSW<-1/(K*(m-1)-1) * (sum(botharm_totals) - sum(botharm_totals_sq)/m)

ICC_detected<-(MSB-MSW)/(MSB+(m*(K-2)/(K-1)-1)*MSW)

deff_detected<-1+(m-1)*ICC_detected

# ICC calculation for true cases

botharm_allcases_sq<-botharm_allcases^2

# Covariance-adjusted ANOVA estimator of ICC for true cases

MSB<-1/(K-1) * (sum(botharm_allcases_sq)/m - sum(botharm_allcases)^2/(m*K))

MSW<-1/(K*(m-1)-1) * (sum(botharm_allcases) - sum(botharm_allcases_sq)/m)

ICC_true<-(MSB-MSW)/(MSB+(m*(K-2)/(K-1)-1)*MSW)

deff_true<-1+(m-1)*ICC_true

# Vaccine effect estimate

VEest<-(1-SAR[1]/SAR[2])*100

sd_ve<-sqrt((1/(SAR[1]*m*nsim) + 1/(SAR[2]*m*nsim) - 2/(nsim*m))*deff_detected)

ci_lower<-1-(1-VEest/100)*exp(1.96*sd_ve)

ci_upper<-1-(1-VEest/100)*exp(-1.96*sd_ve)

trueVEest<-(1-trueSAR[1]/trueSAR[2])*100

truesd_ve<-sqrt((1/(trueSAR[1]*m*nsim) + 1/(trueSAR[2]*m*nsim) - 2/(nsim*m))*deff_true)

trueci_lower<-1-(1-trueVEest/100)*exp(1.96*truesd_ve)

trueci_upper<-1-(1-trueVEest/100)*exp(-1.96*truesd_ve)

cat("\n Estimated efficacy based on SAR: ",VEest,"%\n")

cat ("Total sample size uncorrected for clustering: ",ssu," rings of ",m," persons\n")

cat("sample size corrected (approximately)",deff_detected*ssu,"\n")

cat("Approximate CI for VE: (",ci_lower,",",ci_upper,")\n")

list(out1=VEest,out2=ci_lower,out3=ci_upper,out4=ssu,out5=deff_detected,out6=SAR,out7=ICC_detected,

out8=trueVEest,out9=trueci_lower,out10=trueci_upper,out11=truessu,out12=deff_true,out13=trueSAR,out14=ICC_true)

}

# A function to output multiple outcomes from a function

list <- structure(NA,class="result")

"[<-.result" <- function(x,...,value) {

args <- as.list(match.call())

args <- args[-c(1:2,length(args))]

length(value) <- length(args)

for(i in seq(along=args)) {

a <- args[[i]]

if(!missing(a)) eval.parent(substitute(a <- v,list(a=a,v=value[[i]])))

}

x

}

# Set parameters

pdhosp<-0.2

VEmax<-0.7

startcount<-16

Reff<-0.61

extfoi_numerator<-0

vuptake<-1

ramp_up<-6

pepve<-0

window_length<-21

admin_delay<-0

# Simulation parameters: N is the number of trials to run at the correct sample size,

# to estimate vaccine effect and confidence interval. nsim is the number of rings to run

# to estimate the required sample size. m is the size of each ring

N<-2

nsim<-100

m<-50

# Initialize data frame for results

str<-rep(0,1)

piso<-rep(0,1)

sto<-rep(0,1)

admindelay<-rep(0,1)

vem<-rep(0,1)

veest<-rep(0,1)

veest_ci_lower<-rep(0,1)

veest_ci_upper<-rep(0,1)

veest_med<-rep(0,1)

sar<-rep(0,1)

ssc<-rep(0,1)

ss<-rep(0,1)

icc<-rep(0,1)

cases<-rep(0,1)

ves<-rep(0,1)

daystart<-rep(0,1)

res<-data.frame(piso,str,sto,admindelay,vem,veest,veest_ci_lower,

veest_ci_upper,veest_med,

ss,ssc,icc,sar,cases)

res_perfectasc<-data.frame(piso,str,sto,admindelay,vem,veest,veest_ci_lower,

veest_ci_upper,veest_med,

ss,ssc,icc,sar,cases)

list[VEest,ci_lower,ci_upper,ssu,deff_detected,SAR,ICC_detected,

trueVEest,trueci_lower,trueci_upper,truessu,deff_true,trueSAR,ICC_true]<-

simulatetrial(pdhosp,VEmax,startcount,nsim,Reff,extfoi_numerator,admin_delay,ramp_up,pepve,vuptake,window_length)

res$str[1]<-startcount+admin_delay

res$sto[1]<-startcount+admin_delay+window_length

res$admindelay[1]<-admin_delay

res$vem[1]<-VEmax

res$veest[1]<-VEest/100

res$ss[1]<-ssu

res$ssc[1]<-ssu*deff_detected

res$cases[1]<-mean(SAR)*ssu*deff_detected*m

res$piso[1]<-pdhosp

res$icc[1]<-ICC_detected

res$sar[1]<-mean(SAR)

res_perfectasc$str[1]<-startcount

res_perfectasc$sto[1]<-startcount+window_length

res_perfectasc$admindelay[1]<-admin_delay

res_perfectasc$vem[1]<-VEmax

res_perfectasc$veest[1]<-trueVEest/100

res_perfectasc$ss[1]<-truessu

res_perfectasc$ssc[1]<-truessu*deff_true

res_perfectasc$cases[1]<-mean(trueSAR)*truessu*deff_true*m

res_perfectasc$piso[1]<-pdhosp

res_perfectasc$icc[1]<-ICC_true

res_perfectasc$sar[1]<-mean(trueSAR)

cases_imm<-rep(0,N)

cases_del<-rep(0,N)

veests<-rep(0,N)

pvals<-rep(0,N)

for (trial in 1:N) {

list[veest,,,,,SAR]<-simulatetrial(pdhosp,VEmax,startcount,

ceiling(ssu*deff_detected),Reff,extfoi_numerator,admin_delay,ramp_up,pepve,vuptake,window_length)

cases_imm[trial]<-SAR[1]*m*ceiling(ssu*deff_detected)

cases_del[trial]<-SAR[2]*m*ceiling(ssu*deff_detected)

veests[trial]<-veest

}

veest_median<-median(veests)

cases_imm_median<-median(cases_imm)

cases_del_median<-median(cases_del)

sigma<-sqrt((1/cases_imm_median + 1/cases_del_median - 2/(m*ceiling(ssu*deff_detected)))*deff_detected)

res$veest_med[1]<-veest_median/100

res$veest_ci_lower[1]<-1-(1-veest_median/100)*exp(1.96*sigma)

res$veest_ci_upper[1]<-1-(1-veest_median/100)*exp(-1.96*sigma)

for (trial in 1:N) {

list[,,,,,,,trueveest,,,,,trueSAR

,]<-simulatetrial(pdhosp,VEmax,startcount,

ceiling(truessu*deff_true),Reff,extfoi_numerator,admin_delay,ramp_up,pepve,vuptake,window_length)

cases_imm[trial]<-trueSAR[1]*m*ceiling(truessu*deff_true)

cases_del[trial]<-trueSAR[2]*m*ceiling(truessu*deff_true)

veests[trial]<-trueveest

}

veest_median<-median(veests)

cases_imm_median<-median(cases_imm)

cases_del_median<-median(cases_del)

sigma<-sqrt((1/cases_imm_median + 1/cases_del_median - 2/(m*ceiling(truessu*deff_true)))*deff_true)

res_perfectasc$veest_med[1]<-veest_median/100

res_perfectasc$veest_ci_lower[1]<-1-(1-veest_median/100)*exp(1.96*sigma)

res_perfectasc$veest_ci_upper[1]<-1-(1-veest_median/100)*exp(-1.96*sigma)

write.table(res,paste("results.txt",sep=""),sep="\t")

write.table(res_perfectasc,paste("results_perfectasc.txt",sep=""),sep="\t")
