# Supplementary figures and images for: Using simulation to aid trial design: Ring-vaccination trials
Source: PLoS Negl Trop Dis. 2017 Mar 22;11(3):e0005470. doi: 10.1371/journal.pntd.0005470 (PMC5378415; doi:10.1371/journal.pntd.0005470)

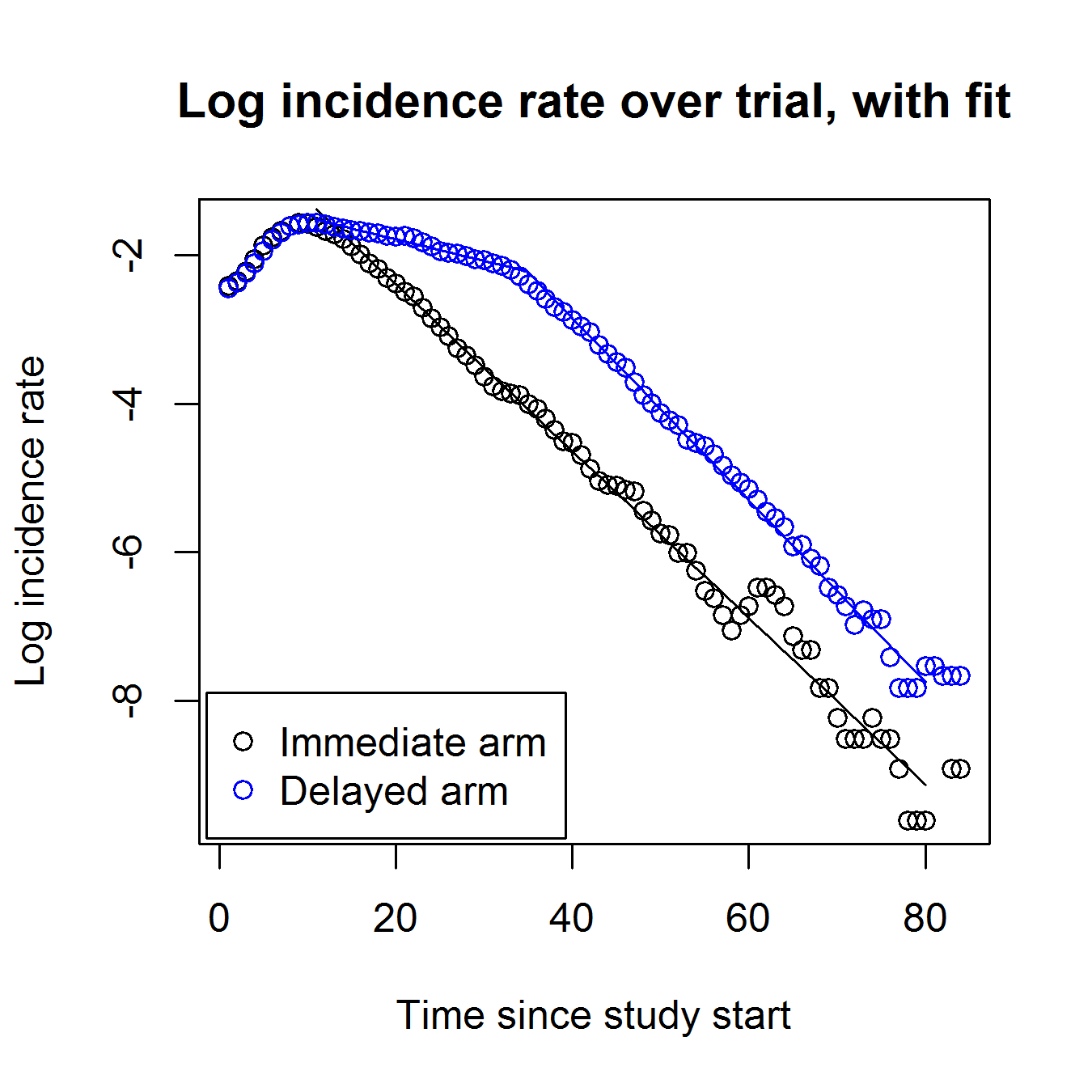

Supplement: S1 Fig — Simulated log incidence rate of detected disease in the trial, in the immediate arm (black circles) and delayed arm (blue circles), with linear fit in the immediate arm (black line) and piecewise linear fit in the delayed arm (blue line). The change in rate in the delayed arm corresponds to the direct effect of the vaccine. Circles represent means over 15,000 simulations. (TIFF) [file pntd.0005470.s003.tiff]

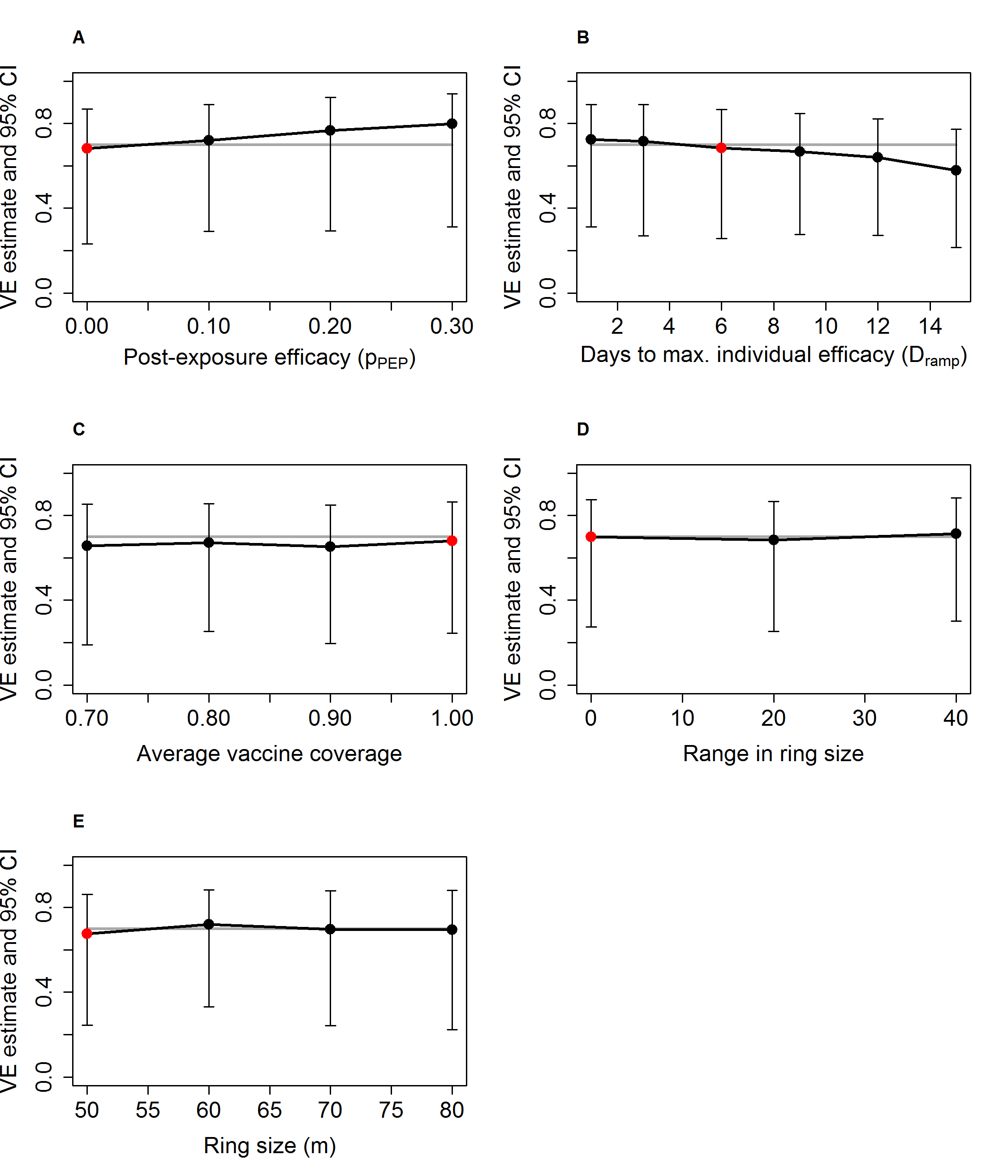

Supplement: S2 Fig — Median point estimate of vaccine effect and 95% confidence interval derived from 100 trials with 80% power to detect vaccine effect shown against: (left to right, top to bottom) A: post-exposure vaccine efficacy, B: days to maximum individual vaccine efficacy, C: average vaccine coverage in a ring, D: range in ring size, and E: ring size. In each panel, the VE estimate corresponding to the baseline parameter set is highlighted in red, and the grey line represents the individual vaccine efficacy of 70%. All other parameters are set at the baseline values. (TIFF) [file pntd.0005470.s004.tiff]

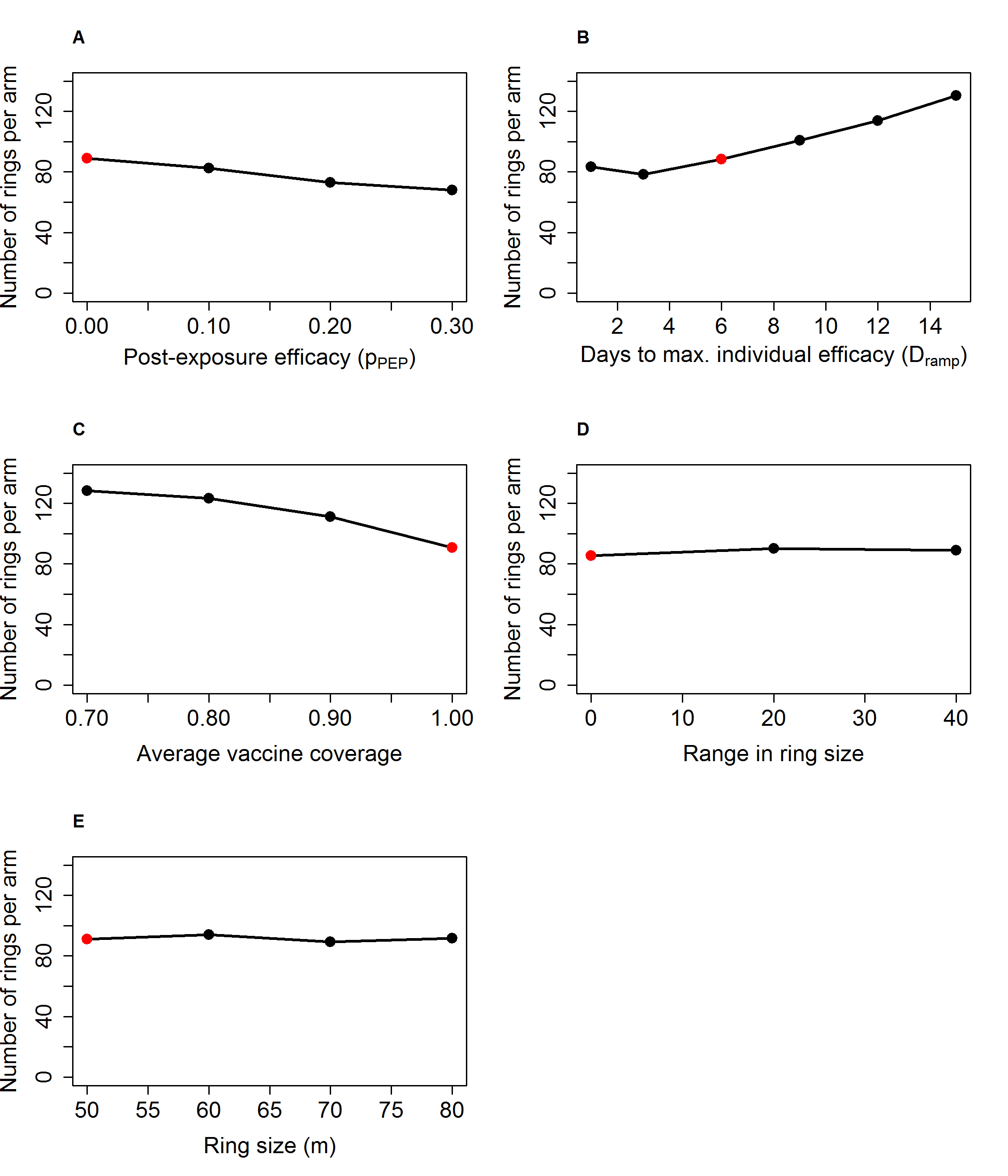

Supplement: S3 Fig — Number of rings per arm required to achieve 80% power to detect a difference in cumulative incidence between the two arms against: (left to right, top to bottom) A: post-exposure vaccine efficacy, B: days to maximum individual vaccine efficacy, C: average vaccine coverage in a ring, D: range in ring size, and E: ring size. In each panel, the sample size estimate corresponding to the baseline parameter set is highlighted in red. All other parameters are set at the default values. (TIFF) [file pntd.0005470.s005.tiff]

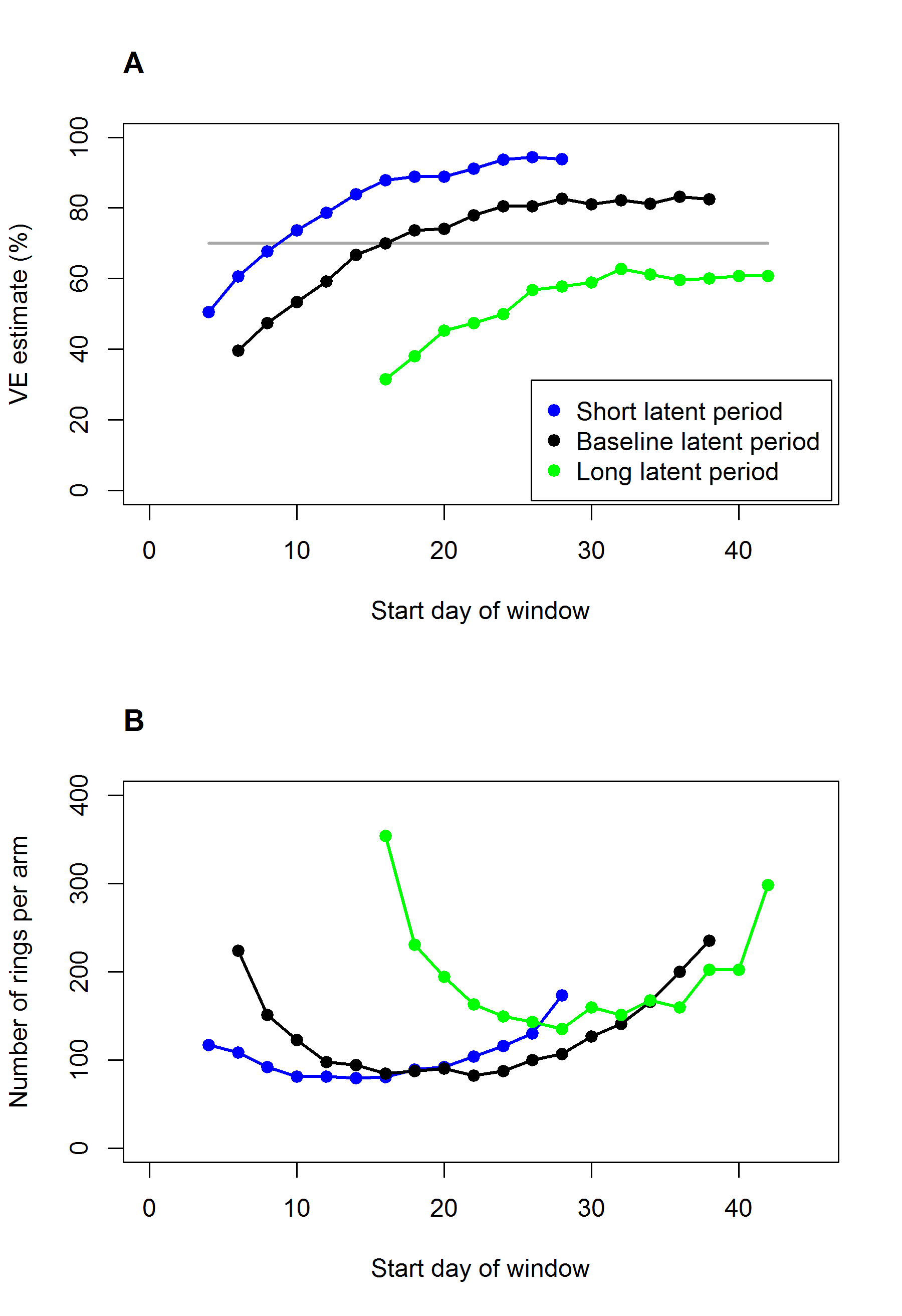

Supplement: S4 Fig — Relationship between the start day of case-counting window and A: the median point estimate of vaccine effect derived from 100 trials with 80% power to detect vaccine effect, and B: required sample size for 80% power to detect vaccine effect, for a disease with a short, baseline and long latent period. In Fig S4A, the grey line represents the individual vaccine efficacy of 70%. All other parameters are set at the baseline values. (TIFF) [file pntd.0005470.s006.tiff]

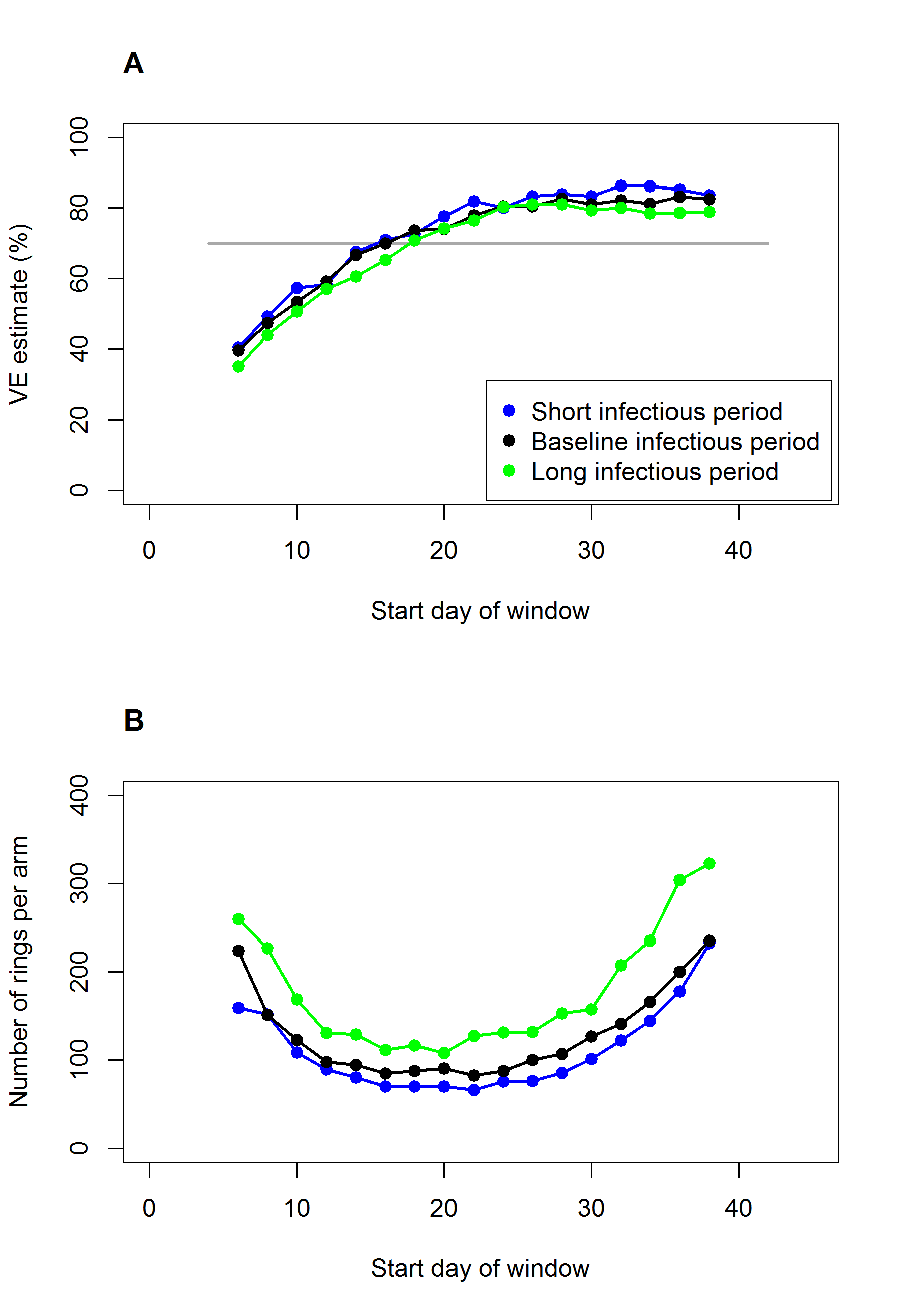

Supplement: S5 Fig — Relationship between the start day of case-counting window and A: the median point estimate of vaccine effect derived from 100 trials with 80% power to detect vaccine effect, and B: required sample size for 80% power to detect vaccine effect, for a disease with a short, baseline and long infectious period. In Fig S5A, the grey line represents the individual vaccine efficacy of 70%. All other parameters are set at the baseline values. (TIFF) [file pntd.0005470.s007.tiff]
